# Case Report of Isoniazid-Related Acute Liver Failure Requiring Liver Transplantation

**DOI:** 10.3390/diseases6020040

**Published:** 2018-05-19

**Authors:** Andrew A. Li, Pratima Dibba, George Cholankeril, Donghee Kim, Aijaz Ahmed

**Affiliations:** 1 Department of Medicine, Stanford University School of Medicine, Palo Alto, CA 94304, USA; andrewli@stanford.edu; 2 Division of Gastroenterology, Women & Infants Hospital/Warren Alpert School of Medicine, Brown University, Providence, RI 02905, USA; pratima_dibba@brown.edu; 3 Division of Gastroenterology and Hepatology, Stanford University School of Medicine, Palo Alto, CA 94304, USA; georgetc@stanford.edu (G.C.); dhkimmd@stanford.edu (D.K.)

**Keywords:** latent tuberculosis infection, INH, isoniazid, INH-related hepatotoxicity, liver transplantation, isoniazid-related hepatotoxicity

## Abstract

The prevalence of latent tuberculosis infection (LTBI) in the United States in 2011 and 2012 was estimated at 4.4–4.8%. As of 2015, 12.4 million people still possessed LTBI. Isoniazid, or isonicotinic acid hydrazine (INH), is the most commonly used medication among varying regimens that exist in the treatment of tuberculosis and LTBI. INH-related hepatotoxicity is a well-known adverse effect of its use, often causing asymptomatic elevations in serum aminotransferase levels. These elevations are typically transient and reversible, but can cause acute, clinically-significant liver injury in rare cases. We report a case of a 67-year old male who developed subacute hepatic injury secondary to INH treatment for LTBI, and ultimately underwent liver transplantation due to the progression to hepatic decompensation, despite withdrawal of the medication. Because symptoms of INH hepatotoxicity are nonspecific and prognosis can be variable, clinicians must maintain a high index of suspicion for this adverse effect. As exemplified by this case, early recognition may be life-saving.

## 1. Introduction

In the United States, individuals with latent tuberculosis infection (LTBI) represent a reservoir of patients whose disease process can progress to tuberculosis (TB) [1]. The prevalence of LTBI in the United States between 2011 and 2012 was estimated at 4.4–4.8% [2]. As of 2015, 12.4 million people still possess LTBI, most of whom are foreign-born, and for whom the prevalence has increased in comparison to American-born patients [2]. Several anti-tuberculosis drug regimens exist for the treatment of tuberculosis and LTBI, most commonly including the use of isoniazid or isonicotinic acid (INH) [3,4]. Some of these drugs, however, have known adverse effects and potency that require close monitoring [5]. Anti-tuberculosis drug-induced hepatotoxicity (ATDH), in particular, has been recognized as its own entity within types of drug-induced liver injury (DILI). While rifampicin and pyrazinamide have been named along with INH as culprits in ATDH, ethambutol and streptomycin have not had the same adverse effects profile [5]. INH-related hepatotoxicity is a well-known adverse effect of the drug’s use, often causing asymptomatic elevations in serum aminotransferase levels [6,7,8]. These elevations are typically transient and reversible, but in some cases can cause acute, clinically-significant liver injury [6,7,8]. In fact, a recent literature review of cases from 1994–2015 identified at least eight other cases of fulminant hepatic failure requiring liver transplantation that were attributed to INH, singly or in combination with other drugs [9]. One study, which focused on adherence to existing American Thoracic Society (ATS) guidelines regarding isoniazid hepatoxicity and reports to the Centers for Disease Control’s (CDC) isoniazid adverse events program, demonstrated that poor adherence to the guidelines is common and results in late recognition and severe liver injury [10]. Therefore, patients who are treated with INH should be counseled regarding the possibility of hepatitis, screened for susceptibility based on the presence of certain risk factors, and monitored for signs and symptoms of such so as to withdraw the medication. Despite such interventions, however, there is still a rare risk of progression to fulminant hepatic failure.

We report a case of a 67-year old man who developed subacute hepatic injury secondary to INH treatment for LTBI, who ultimately required liver transplant surgery due to the progression to fulminant hepatic failure despite withdrawal of the medication.

## 2. Case Report

A 67-year-old man from India, who was recently diagnosed with latent tuberculosis, presented to an outside hospital and was admitted for six days with generalized fatigue and hypotension. On presentation, he reported a 2-week history of generalized fatigue with systolic blood pressures in the 60s, as recorded at home. He also reported a 30-pound weight loss which was attributed to diuretic use and dietary changes in the setting of congestive heart failure. With these complaints, his losartan (angiotensin receptor blocker) dose was reduced, which was ineffective in alleviating his symptoms. His history was notable for an LTBI diagnosed three months prior to presentation, for which he was being treated with INH (300 mg daily) and pyridoxine. He had been treated for 11 weeks by the time he presented, and was noted to tolerate the therapy well for at least four weeks without changes in dietary or sleeping habits, per documentation by his primary care provider. His medical history was otherwise notable for atrial fibrillation, beta thalassemia, and tachycardia-induced cardiomyopathy with reduced ejection fraction. He had no known history of liver disease or diabetes. His other medications included apixiban, metoprolol, furosemide, losartan, and hydroxyzine (for insomnia). He had no prior history of heavy alcohol consumption or recreational drug use, and he worked in the electronics and computer industry. There was no family history of cirrhosis or other liver disease.

His initial workup was notable for elevated liver function tests, as shown in Table 1. A computed tomography scan of the abdomen demonstrated no intrahepatic biliary dilation and no apparent fatty change. An abdominal ultrasound revealed a simple cyst and heterogenous echotexture, with mild subcapsular nodularity. Based on his workup, his liver dysfunction was postulated to be drug-induced liver injury from INH or apixaban. Both medications were discontinued. He was discharged for outpatient management. However, follow-up laboratory testing indicated an increasing bilirubin, which resulted in readmission to the hospital. A liver biopsy was considered but not performed, as his bilirubin levels began to downtrend.

One week later, the patient experienced a syncopal episode while having a bowel movement, and was admitted to a different hospital. He presented with altered mental status and generalized pruritis. Laboratory data revealed worsening liver function (Table 1) and acute kidney injury, for which he was treated with albumin, midodrine, and octreotide. His pruritis improved with the introduction of cholestyramine.

Autoimmune serologies, including antinuclear antibody, antimitchondrial antibody, and anti-smooth muscle antibody, were negative. Viral hepatitis serologies were negative. HIV infection was ruled out. The patient’s acetaminophen level, urine toxicology screen, and serum ferritin level were within normal limits. A transjugular liver biopsy was performed and demonstrated cholestatic hepatitis, thought to be drug- or toxin- related. His mental status initially improved with lactulose, but worsened thereafter, prompting a transfer to our hospital for consideration of liver transplantation for acute liver failure, about six weeks after initial presentation.

Physical examination at time of transfer was remarkable for jaundice, icteric sclera, and altered mental status. He was somnolent but arousable to noxious stimuli, oriented only to self, intermittently following some commands, and had notable asterixis. He had an irregular rhythm and a murmur on cardiac examination, vesicular breath sounds, and a benign abdominal examination. He was noted to have Grade III hepatic encephalopathy in the setting of acute liver failure, and was admitted to the intensive care unit before being listed for liver transplantation as Status 1A. On day four in the hospital, he underwent orthotopic liver transplant surgery, and was extubated on post-operative day one. He was transferred to the floor on post-operative day three, but subsequently suffered a sudden aspiration event resulting in a pulseless electrical activity and fatal cardiopulmonary arrest.

## 3. Discussion

DILI is one of the most common causes of liver dysfunction [11]. INH, in addition to amoxicillin/clavulanate and non-steroidal anti-inflammatory drugs, are the most commonly named etiologies for DILI [12]. DILI is defined by injury to the liver, as caused by a drug, which can be an over-the-counter medication, herbal supplement, or prescribed medication [12]. DILI can be classified as hepatitic (or hepatocellular), cholestatic, or mixed, based upon liver biochemical criteria—particularly the *R* ratio. It represents the ratio of alanine aminotransferase to the alkaline phosphatase, with respect to their upper limits of normal [12]. To meet criteria for hepatitic DILI, the ratio of alanine transaminase (ALT) to alkaline phosphatase, both expressed as multiples of upper limit, or the *R* ratio, should be greater than or equal to five, with ALT greater than or equal to three times the upper limit of normal [12]. A cholestatic picture of DILI typically possesses a ratio of ALT to alkaline phosphatase of less than two, with alkaline phosphatase being two times the upper limit of normal [12]. Mixed DILI presents with an overall ALT to alkaline phosphatase ratio between two and five, with ALT being greater than three times the upper limit of normal, and alkaline phosphatase at two times the upper limit of normal [12]. Such formulas are limited by the paucity of data regarding the particular time frame by which DILI evolves [12].

DILI can also be categorized as immune or non-immune, based on the presence of systemic features and onset of symptoms [12]. Immune DILI is associated with an allergic reaction or hypersensitivity reaction, and is early in onset, with rapid re-injury observed when the drug is reintroduced [12]. Non-immune-mediated DILI lacks systemic features, has a later onset of action, and does not cause the same rapid re-injury observed in the former [12]. INH has previously been categorized as non-immune, although more recent literature suggests that the metabolites of INH can elicit an immune response and liver injury, particularly in those patients who have the phenotype associated with the production of anti-drug/anti-CYP P450 antibodies [13]. Additionally, DILI can be categorized as either intrinsic or idiosyncratic [14]. Intrinsic DILI is seen in drugs that predictably cause injury in high doses, such as acetaminophen [14]. However, idiosyncratic DILI, exhibited by INH, is seen in certain susceptible individuals with less dose-related but highly variable patterns [14]. Susceptibility is based on the presence of certain risk factors, which will be discussed later.

DILI is a diagnosis of exclusion, with the primary tool for diagnosis being a history and physical exam [14]. Blood tests and imaging studies are then tailored to the particular clinical scenario [14]. Of note, hepatic enzymes can be used to calculate the aforementioned *R* ratio. The differential remains can be ruled out with additional testing; such diagnoses include viral hepatitis, Wilson’s disease, Budd-Chiari syndrome, ischemic liver injury, and autoimmune hepatitis [14]. The most clinically suspicious differential diagnoses in our patient were excluded by laboratory testing and liver biopsy. A liver biopsy is strongly recommended to differentiate autoimmune hepatitis from DILI when the clinical scenario is unclear. Although biopsies are not mandatory for diagnosis of DILI, histologic findings can be observed with different drugs [12]. INH hepatotoxicity histologically resembles viral hepatitis, but can exhibit additional findings, such as increased eosinophils and cholestasis, as observed in our patient.

The onset of INH hepatotoxicity typically begins between weeks 2 and 24. Patients present with nonspecific symptoms, including, nausea, anorexia, jaundice, abdominal pain, weakness, fatigue, and easy bruising [5,9]. Symptomatic elevations of transaminases are noted in 10–20% of patients receiving INH [15]. Our patient presented classically, with an *R* ratio consistent with a hepatitic DILI, and was symptomatic. Like our patient, fatality can be as high as 10% in those with clinically evident hepatitis secondary to INH use [15]. Typically, withdrawal of the offending agent can reverse the damage induced in DILI [14]. While some experts consider ruling out the diagnosis of DILI when there is a lack of improvement in peak ALT levels by 50% within 30 days of withdrawal of the offending agent, there is rare (but existent) literature suggesting that drugs like INH can rapidly progress to liver failure despite withdrawal, as in our case report [14]. The pathophysiology behind INH hepatotoxicity has been hypothesized to be related to the rapid metabolism of INH in susceptible individuals [6]. Specifically, INH’s mechanism of injury is thought to be a result of the accumulation of toxic metabolic intermediates, although more recent studies implicate the polymorphism of *N*-acetyltransferase 2 and glutathione-*S*-transferase as major susceptibility risk factors [16]. In one study, patients with a rapid acetylator phenotype hydrolyzed larger amounts of INH to isonicotinic acid and hydrazine, which is liberated from INH and has been found to produce liver necrosis in animal studies [6]. Studies in animals and humans have shown that hydrazine is a toxic metabolite capable of causing hepatic steatosis, hepatocyte vacuolation, and glutathione depletion [5]. Other studies found that slow acetylators develop more severe ATDH, due to the accumulation of metabolites that convert to hydrazine [5]. INH has also been shown to influence cytochrome P450 enzymes, inducing its own toxicity [5]. Known risk factors that make an individual susceptible to INH hepatotoxicity include advanced age, female gender, alcohol use, cirrhosis or other pre-existing liver disease, having an Asian racial background, chronic viral hepatitis, slow acetylator status, and concurrent use of other known potential hepatotoxic medications [17]. Our patient had several of the aforementioned risk factors, making him susceptible to INH hepatotoxicity and classic for idiosyncratic DILI.

Current guidelines proposed by the ATS, the CDC, and the Infectious Diseases Society of America suggest that patients receiving INH undergo a monthly clinical evaluation to screen for adverse events, with closer surveillance for those with the aforementioned risk factors [18]. Transaminases should be obtained prior to therapy and every four weeks during use, particularly in those with risk factors or with abnormal baseline liver enzymes [18]. For confirmed cases of INH hepatotoxicity or ATDH in general, culprits should ideally be discontinued and reintroduced once hepatotoxicity has resolved. In low-income countries, where liver functions tests are not available to confirm the diagnosis, treatment is based on symptoms [5]. Drug therapy should be discontinued at any sign or symptom of hepatotoxicity, and resumed two weeks after symptoms have resolved [5]. In the case of our patient, withdrawal of the agent despite confirmatory testing was futile.

## 4. Conclusions

We report a case of severe INH hepatotoxicity that progressed despite withdrawal of the drug, ultimately requiring liver transplantation for acute liver failure six weeks after the patient’s initial presentation of decompensated liver disease. Post-operatively, the patient’s encephalopathy improved, although the patient later suffered death from aspiration and cardiopulmonary arrest. While discontinuation of INH improves hepatotoxicity in most cases, this particular case is representative of the rare occurrence of the progression of liver disease to fulminant liver failure, despite withdrawal of the offending agent. This patient admittedly possessed risk factors that predisposed him to a worse outcome, including age and the use of other potentially hepatotoxic agents. His clinical scenario was also confounded by his non-specific symptoms, which were presumed to be associated with his other co-morbidities.

Because symptoms of INH hepatotoxicity are nonspecific and prognosis can vary per case, clinicians must maintain a high index of suspicion for this adverse effect. Patients receiving INH who report symptoms that may correlate with INH hepatotoxicity, or who possess risk factors that may increase risk of the adverse effect, should be evaluated for hepatotoxicity with biochemical testing, and therapy should be withdrawn. Alternate regimens and expert consultation should be considered, depending upon the outcome for the patient [18].

## Figures and Tables

**Table 1:** Selected laboratory values from prior baseline, prior congestive heart failure (CHF) admission, and multiple admissions related to isonicotinic acid hydrazine (INH) hepatotoxicty.

Comments	11/5/16	6/3/17	6/13/17	6/24/17	7/6/17	7/11/17	7/13/17	7/16/17	7/18/17
	Baseline Pre-INH	Initial Admission	Second Admission	Third Admission			Transfer	Pre-OLT	POD1
Total bilirubin (mg/dL)	1.6	20.7	39.6	43.9	55.6	48.1	48	48	12.1
Direct bilirubin (mg/dL)	0.2	15.9	34			29.3	31.3		
AST (U/L)	23	757	258	227	261	244	253	336	370
ALT (U/L)	22	964	273	188	175	178	204	229	237
Alkaline phosphatase (U/L)	76	171	166	100	56	58	87	108	49
Albumin (g/dL)	4	3.3	2.6	2.4	4	3	3.2	3	1.8
Sodium (mmol/L)	140	130	134	127	145	140	138	153	145
Creatinine (mg/dL)	1.1	1.4	1.2	1.6	1.1	1.1	0.81	0.84	2.6
INR		2.8	1.7	2.2	2.6		2.8	2.6	1.7
MELD-Na		33	30	36	33		33	32	
Ammonia (umol/L)				51	28	124			

OLT: orthotopic liver transplant; POD1: post-operative day 1; INR: international normalized ratio; MELD-Na: Model for End Stage Liver Disease with sodium correction

## Abbreviations

LTBI	Latent tuberculosis infection
TB	Tuberculosis
INH	Isoniazid or isonicotinic acid
ATDH	Antituberculosis drug-induced hepatotoxicity
DILI	Drug-induced liver injury

## References

[1] Horsburgh C.R., Rubin E.J. (2011). Latent Tuberculosis Infection in the United States. N. Engl. J. Med..

[2] Mancuso J.D., Diffenderfer J.M., Ghassemieh B.J., Horne D.J., Kao T.C. (2016). The prevalence of latent tuberculosis infection in the United States. Am. J. Respir. Crit. Care Med..

[3] American Thoracic Society, Centers for Disease Control, Infectious Diseases Society of America (2000). Targeted tuberculin testing and treatment of latent tuberculosis infection. Am. J. Respir. Crit Care Med..

[4] Centers for Disease Control and Prevention (CDC) (2011). Recommendations for Use of an Isoniazid-Rifapentine Regimen with Direct Observation to Treat Latent Mycobacterium tuberculosis Infection. Morb. Mortal. Wkly. Rep..

[5] Tostmann A., Boeree M.J., Aarnoutse R.E., De Lange W.C.M., Van Der Ven A.J.A.M., Dekhuijzen R. (2008). Antituberculosis drug-induced hepatotoxicity: Concise up-to-date review. J. Gastroenterol. Hepatol..

[6] Mitchell J.R., Zimmerman H.J., Ishak K.G., Thorgeirsson U.P., Timbrell J.A., Snodgrass W.R., Nelson S.D. (1976). Isoniazid liver injury: Clinical spectrum, pathology, and probable pathogenesis. Ann. Intern. Med..

[7] Byrd R.B., Horn B.R., Solomon D.A., Griggs G.A. (1979). Toxic effects of isoniazid in tuberculosis chemoprophylaxis: Role of biochemical monitoring in 1000 patients. JAMA.

[8] Nolan C.M., Goldberg S.V., Buskin S.E. (1999). Hepatotoxicity associated with isoniazid preventive therapy: A 7-year survey from a public health tuberculosis clinic. JAMA.

[9] Li X., Liu Y., Zhang E., He Q., Tang Y.-B. (2015). Liver Transplantation in Antituberculosis Drugs-Induced Fulminant Hepatic Failure: A Case Report and Review of the Literature. Medicine.

[10] Hayashi P.H., Fontana R.J., Chalasani N.P., Stolz A.A., Talwalkar J.A., Navarro V.J., Hoofnagle J.H. (2015). Under-reporting and poor adherence to monitoring guidelines for severe cases of isoniazid hepatotoxicity. Clin. Gastroenterol. Hepatol..

[11] Zimmerman H.J. (2000). Drug-induced liver disease. Clin. Liver Dis..

[12] Leise M.D., Poterucha J.J., Talwalkar J.A. (2014). Drug-induced liver injury. Mayo Clin. Proc..

[13] Metushi I., Uetrecht J., Phillips E. (2016). Mechanism of isoniazid-induced hepatotoxicity: Then and now. Br. J. Clin. Pharmacol..

[14] Chalasani N.P., Hayashi P.H., Bonkovsky H.L., Navarro V.J., Lee W.M., Fontana R.J. (2014). ACG Clinical Guideline: The diagnosis and management of idiosyncratic drug-induced liver injury. Am. J. Gastroenterol..

[15] Murray K.F., Hadzic N., Wirth S., Bassett M., Kelly D. (2008). Drug-related hepatotoxicity and acute liver failure. J. Pediatr. Gastroenterol. Nutr..

[16] Hussain Z., Ka P., Husain S.A. (2003). Antituberculosis drug-induced hepatitis: Risk factors, prevention and management. Indian J. Exp. Biol..

[17] Saukkonen J.J., Cohn D.L., Jasmer R.M., Peloquin C.A., Gordin F.M., Nunes D., Strader D.B., Bernardo J., Venkataramanan R., Sterling T.R. (2006). An official ATS statement: Hepatotoxicity of antituberculosis therapy. Am. J. Respir. Crit. Care Med..

[18] Nahid P., Dorman S.E., Alipanah N., Barry P.M., Brozek J.L., Cattamanchi A., Higashi J.M. (2016). Official American Thoracic Society/centers for disease control and prevention/infectious diseases society of America clinical practice guidelines: Treatment of drug-susceptible tuberculosis. Clin. Infect. Dis..

